# Prevalence, risk factors and treatment outcomes of isoniazid resistant TB in Bulawayo city, Zimbabwe: A cohort study

**DOI:** 10.3855/jidc.12319

**Published:** 2020-08-31

**Authors:** Barbara Manyame-Murwira, Kudakwashe Collin Takarinda, Pruthu Thekkur, Bright Payera, Herbert Mutunzi, Raiva Simbi, Nicholas Siziba, Edwin Sibanda, Catherine Banana, Norbert Muleya, Evidence Makombe, Paula Littia Jongwe, Regina Bhebhe, Douglas Mangwanya, Janet Dzangare, Fungai H Mudzengerere, Collins Timire, Enock Wekiya, Charles Sandy

**Affiliations:** 1 National Tuberculosis Reference Laboratory, Ministry of Health and Child Care, Bulawayo, Zimbabwe; 2 AIDS and TB Department, Ministry of Health and Child Care, Harare, Zimbabwe; 3 Centre for Operational Research, International Union Against Tuberculosis and Lung Disease, Paris, France; 4 The Union South-East Asia (The USEA) Office, New Delhi, India; 5 Department of Laboratory Services, Ministry of Health and Child Care, Harare, Zimbabwe; 6 Department of Health, Bulawayo City Council, Zimbabwe; 7 Department of Environmental Health Services, Ministry of Health and Childcare, Matebeleland South, Zimbabwe; 8 Department of laboratory Services, Ministry of Health and Childcare, Midlands Province, Zimbabwe; 9 Family Health International (FHI360), Harare, Zimbabwe; 10 WHO Supra National Reference Laboratory/National Tuberculosis Reference Laboratory, Uganda

**Keywords:** Tuberculosis, isoniazid mono-resistance, IPT, Operational Research, SORT-IT

## Abstract

**Introduction:**

The isoniazid-resistant TB poses a threat to TB control efforts. Zimbabwe, one of the high TB burden countries, has not explored the burden of isoniazid resistant TB. Hence among all bacteriologically-confirmed TB patients diagnosed in Bulawayo City during March 2017 and December 2018, we aimed to assess the proportion with isoniazid resistant TB and associated factors. Also, we aimed to describe the TB treatment outcomes.

**Methodology:**

A cohort study involving routinely collected data by the National TB Reference Laboratory (NTBRL) in Bulawayo City and National TB programme of Zimbabwe. The percentage with 95% confidence interval (CI) was used to express the proportion with isoniazid-resistant TB. The modified Poisson regression was used to assess the association of demographic and clinical characteristics with isoniazid mono-resistant TB.

**Results:**

Of 2160 bacteriologically-confirmed TB patients, 1612 (74.6%) had their sputum received at the NTBRL and 743 (46.1%) had culture growth. Among those with culture growth, 34 (4.6%, 95% CI: 3.5–6.7) had isoniazid mono-resistant TB, 25 (3.3%, 95% CI: 2.2–4.9) had MDR-TB. Thus, 59 (7.9%, 95% CI: 6.1–10.1) had isoniazid-resistant TB. Children < 15 years had a higher prevalence of isoniazid mono-resistant TB (aPR= 3.93; 95% CI: 1.24–12.45). Among those with rifampicin sensitive TB, patients with isoniazid-sensitive TB had higher favourable treatment outcomes compared to those with isoniazid-resistant TB (86.3% versus 75.5%, p = 0.039).

**Conclusions:**

The prevalence of isoniazid-resistant TB was low compared to neighbouring countries with high burden of TB-HIV. However, Zimbabwe should consider reviewing treatment guidelines for isoniazid mono-resistant TB due to the observed poor treatment outcomes.

## Introduction

Tuberculosis (TB) remains a major public health problem globally, with an estimated 10 million incident cases and an estimated 1.2 million deaths in 2018 [1]. Despite a noticeable decline in the TB burden over the last two decades, the emergence of drug-resistant TB has posed a threat to TB control efforts. In 2013, the World Health Organization (WHO) described resistance to TB drugs as a ‘ticking time bomb’ among called for ‘visionary political leadership’ to mitigate this threat [2].

Isoniazid is an important, effective and cheap first-line anti-TB drug with low rates of adverse events [3]. The drug provides a high initial kill at the start of active TB treatment compared to other first-line TB drugs used in standard anti-TB treatment regimen recommended by WHO [4]. Isoniazid is also used as chemoprophylaxis against TB in selected high-risk groups like people living with HIV (PLHIV) and those with latent TB infection (LTBI) [5].

A recent systematic review reported that resistance to isoniazid could make the standard anti-TB treatment regimen weak and eventually lead to higher rates of unfavourable treatment outcomes [6]. Therefore, WHO recommended the addition of fluoroquinolones in the regimen for managing TB patients with isoniazid resistance [7]. However, countries were directed to adopt an appropriate regimen for TB patients with isoniazid resistance based on the existing prevalence of isoniazid resistance, availability of culture and drug susceptibility testing (CDST) and prevalence of fluoroquinolone resistance [7].

In 2011, a systematic review estimated the global prevalence of isoniazid resistance to be 13.9% among TB patients, with exclusion of Eastern Europe where the prevalence was 44.9% [8]. However, this estimate was unreliable as data were available only from 106 countries. During the review, Botswana and South Africa were the only two high HIV burden countries that had information on isoniazid resistance and both showed an increasing trend [9, 10]. Thus, the review recommended for future research to assess the prevalence of isoniazid resistance in high HIV burden countries [11].

Zimbabwe is a high HIV burden country with a triple burden of TB, TB/HIV and multi-drug resistant TB (MDR-TB). In 2017, there were an estimated 30,000 new TB patients and 4,600 deaths due to TB [1]. Adhering to WHO recommendations for surveillance of anti-TB drug resistance, the national TB programme (NTP) of Zimbabwe carried out the first drug resistance survey in the year 2016. However, the survey failed to estimate the prevalence of isoniazid resistance among TB patients as CDST was done only among those with rifampicin resistance.

The prevalence of isoniazid resistance could not be estimated under routine programme, as the NTP recommended CDST only among rifampicin-resistant TB patients. However, in March 2017, the diagnostic algorithm was revised and CDST was offered to all bacteriologically-confirmed TB patients. Although the algorithm was revised again in September 2017 with the recommendation of CDST reserved for rifampicin-resistant TB patients, the change was partially disseminated to health care providers in the Bulawayo metropolitan province of Zimbabwe. Thus, the health workers from peripheral health facilities of the province continued to send sputum samples of bacteriologically-confirmed TB patients for CDST at the National tuberculosis reference laboratory (NTBRL). This provided a unique opportunity to estimate the burden of isoniazid resistance under routine programmatic settings.

We therefore set out to determine, among bacteriologically confirmed TB patients diagnosed between March –2017 and December-2018 in the in Bulawayo metropolitan province, the number and proportion; 1) undergoing first-line DST 2) showing bacterial growth on culture media, among those who underwent CDST 3) with isoniazid resistance among those with growth on culture and associated factors. Also, to compare the programmatic TB treatment outcomes among the rifampicin sensitive TB patient with and without isoniazid resistance.

## Methodology

### Study Design

We conducted a cohort study using secondary data routinely collected by the NTBRL in Bulawayo and NTP of Zimbabwe.

### Study Setting

#### General Setting

Zimbabwe is a landlocked country in southern Africa with a total population of 16.5 million [12]. The country is divided into ten provinces of which two of them are metropolitan. The majority (67%) of the population resides in rural areas and an estimated 72% of the population is below the poverty line. HIV and TB care services are offered free of charge and are integrated with general health services.

#### Specific Setting

The diagnosis of TB, first-line CDST and treatment of tuberculosis in the Bulawayo metropolitan province of Zimbabwe are described below.

### Diagnosis of bacteriologically confirmed TB

The presumptive pulmonary TB patients identified using the WHO symptom TB screening tool (current cough, fever, haemoptysis, weight loss and night sweats) at health facilities are offered Xpert MTB/Rif assay (Cepheid, Sunnyvale, CA, USA) for diagnosis of TB. Two spot sputum samples (two hours apart) are collected at the facility and are transferred to the GeneXpert site. One of the two sputum samples is subjected to Xpert MTB/Rif assay. If the sample is positive for MTB, the second sample is sent to NTBRL for CDST. Health facilities also send extra-pulmonary specimens for bacteriological confirmation of TB. These specimens are divided into two parts and one part is subjected to Xpert MTB/Rif assay. If MTB is detected, the other part of the specimen is sent to NTBRL for first-line DST.

At the GeneXpert site, the demographic details of the patients, assay result, information on referral for treatment and details of dispatch of the second sputum to NTBRL are documented in the “laboratory register” by the lab technician.

### First-line drug CDST at NTBRL

Samples at the NTBRL are decontaminated using the modified Petroff method and inoculated on both MGIT (liquid) (Becton Dickinson and Co, New Jersey, USA) and Lowenstein-Jensen (solid) culture media. The procedure of decontamination, inoculation and culture are in line with the Stop TB Partnership mycobacteriology laboratory manual [13]. In the samples which show growth, identification of the bacteria is made using the SD Bioline kit (Standard Diagnostics, Inc., Korea) and the isolate is subjected to first-line phenotypic DST. The DST is conducted at a critical concentration of isoniazid at 0.1μg/mL, rifampicin at 1.0μg/mL, ethambutol at 5.0μg/mL and streptomycin at 1.0μg/mL [14].

The external quality assurance panels from the supranational reference laboratory assess the quality of CDST at the NTBRL. The NTBRL was graded excellent for quality during the study reference period.

The details of samples reaching the NTBRL and the CDST results are documented in the electronic CDST database maintained at the NTBRL.

### Tuberculosis treatment

The GeneXpert site informs the result of Xpert MTB/Rif assay to health facilities for patient-tracking and initiation of anti-TB treatment. In the health facilities, all the rifampicin-sensitive TB patients are initiated by the nurse or physician on standard anti-tuberculosis treatment as recommended by the NTP [15].

All the TB patients receive two months of intensive phase (2HRZE: H = Isoniazid, R = Rifampicin, P = Pyrazinamide, E = Ethambutol) and four months of continuation phase (4HR). Fixed-dose combination (FDC) drugs of HRZE (4FDC) and HR (2FDC) are provided. The number of tablets to be consumed ranges from two to five based on the weight of the patient. The staff nurse provides directly observed treatment. Fluoroquinolone based regimens for TB patients with isoniazid resistance is not practiced in Zimbabwe. The healthcare provider ascertains the treatment outcomes of the TB patients as per NTP guidelines [15].

At health facilities, the socio-demographic, clinical and treatment outcome details of TB patients initiated on treatment are documented in the TB register maintained by the staff nurse.

### Study Population

We included all bacteriologically-confirmed TB patients diagnosed between March 2017 and December 2018 in the Bulawayo metropolitan province of Zimbabwe.

### Data Variables, sources of data and data collection

The data variables including patient names, age, gender, type of TB, site of TB and Xpert assay results were obtained from the Gene Xpert site ‘laboratory registers’ between April and May-2019 using a customized Excel database populated by laboratory technicians.

This data was later merged with data from the NTBRL database on receipt of samples at the NTBRL, culture growth and CDST results using patient names, age, gender and the Gene Xpert site. Details on HIV status, antiretroviral therapy (ART) status and TB treatment outcomes were later traced from each patient’s respective DOT health facility and updated into the database.

### Data entry and analysis

The data entered in Microsoft Excel was exported for analysis using Stata (version 15.0 STATA Corporation, USA). Socio-demographic and clinical characteristics of participants were summarized using percentages. The Chi Square test was used to compare the socio-demographic and clinical characteristics of bacteriologically confirmed TB patients with- and without- receipt of samples at the NTBRL. Similar comparison was done among patients with receipt of samples at NTBRL with- and without- culture growth. Multivariate-adjusted prevalence ratios and their respective 95% confidence intervals were calculated using a generalized linear regression model with a log-binomial link to determine factors associated with having isoniazid mono-resistance. The TB treatment outcomes of rifampicin sensitive TB patients were compared between those with and without isoniazid resistance using a Chi Square test. P-values were set 5% levels of significance.

### Ethics

The ethics approval for the study was obtained from the Ethics Advisory Group of the International Union Against Tuberculosis and Lung Disease, Paris, France (40/19) and the Medical Research Council of Zimbabwe (MRCZ/E/255). Permission to access data was obtained from the Ministry of Health and Child Care, Zimbabwe.

## Results

In Bulawayo province during the study reference period, 2160 individuals were diagnosed with bacteriologically-confirmed TB patients. Of the total, the median age was 36 (Interquartile range, 29–45) years and 1314 (60.8%) were males. Of the 2160 patients, 2031 (94.0%) had new TB, 2017 (93.4%) had pulmonary TB and 153 (7.1%) had rifampicin resistance detected on Xpert MTB/Rif assay. Of the 1346 (62.3%) with recorded HIV status, 976 (72.5%) were HIV co-infected, of whom, 741 (75.9%) were receiving ART (Table 1.)

**Figure 1.**
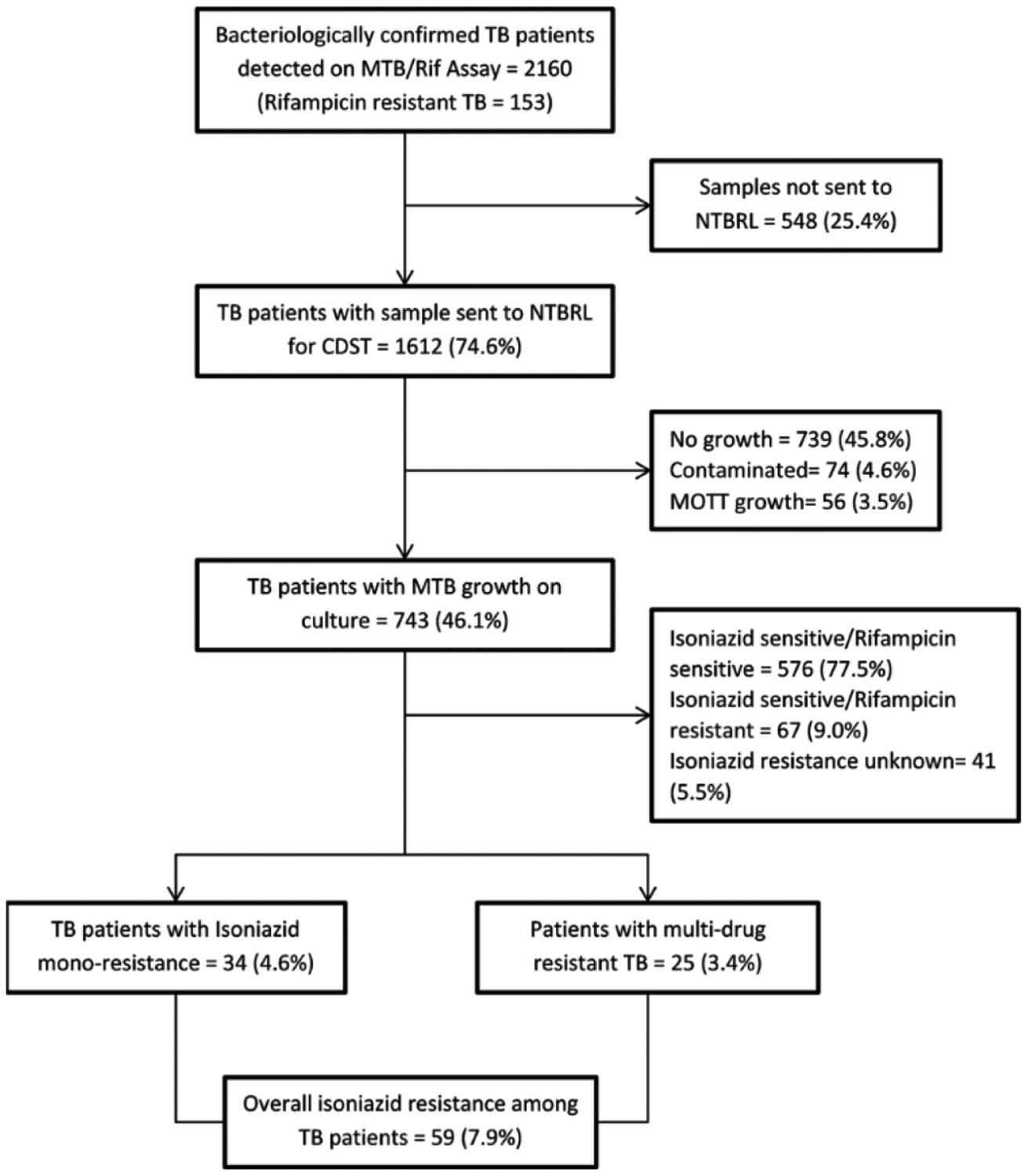
Flow chart of specimen submission to the National TB Reference Laboratory, results of CDST and isoniazid resistance among all bacteriologically-confirmed TB patients in Bulawayo, Zimbabwe (March 2017 – December 2018). * This includes 67 who were Rif resistant; MTB = Mycobacterium tuberculosis; Rif = Rifampicin; NTBRL = National TB Reference laboratory; MOTT- Mycobacterium Other Than Tuberculosis; CDST-Culture and Drug Susceptibility Testing.

Of all the bacteriologically confirmed TB patients, 1612 (74.6%) had their sputum specimen sent to the NTBRL and of these, 743 (46.1%) had culture growths. In those with culture growths, 34 (4.6%, 95% CI: 3.5%−6.7%) had isoniazid mono-resistance and 25 (3.3%, 95%CI: 2.2%−4.9%) had multidrug resistant TB. Overall, 59 (7.9%, 95% CI: 6.1%−10.1%) had isoniazid resistance among those with culture growth (Figure 1).

On comparison of baseline demographic and clinical characteristics of bacteriologically confirmed TB patients with- and without- receipt of samples at the NTBRL, the groups statistically differed in distribution of ‘site of TB’ (p value = 0.013) and ‘HIV/ART status’ (p < 0.001) (Supplementary Table 1).

On comparison of baseline demographic and clinical characteristics of those with- and without-culture growth among those with sample received at NTBRL, age (p = 0.005), gender (p < 0.001), type of TB (p < 0.001), site of TB (p < 0.001) and ‘HIV/ART status’ (p < 0.001) differed statistically across the two groups (Supplementary Table 2).

The demographic and clinical characteristics associated with isoniazid mono-resistance among those with culture growth and rifampicin sensitive TB is shown in Table 2. Those with age < 15 years had higher (aPR = 3.93; 95% CI: 1.24–12.45) prevalence of isoniazid mono-resistance compared to those who were 25–34 years old.

Comparison of TB treatment outcomes between those with- and without- isoniazid resistance among bacteriologically confirmed TB patients with rifampicin sensitive TB are shown in Table 3. Of the 610 patients with rifampicin sensitive TB, 604 (99%) were started and notified on TB treatment. Among these notified TB patients, those with isoniazid resistant TB had poorer TB treatment outcomes in comparison to those with isoniazid sensitive TB (25 (73.5%) versus 492 (86.3%), p = 0.039). Deaths were observed in 5 (14.7%) of the isoniazid resistant TB patients and 37 (6.5%) of isoniazid sensitive TB patients (p = 0.078).

## Discussion

This is the first study from Zimbabwe assessing the extent of isoniazid resistance under the routine national TB programme. There were some findings of note. First, about three-quarters of bacteriologically-confirmed TB patients detected on Xpert MTB/Rif assay, had their sputum specimens received at the NTBRL for CDST. Second, only half of specimens received at the NTBRL had a culture growth despite the culture contamination rate being within the recommended limits. Third, one in thirteen of patients with a culture growth had any isoniazid resistance whilst approximately one in twenty had isoniazid mono-resistance (resistant to isoniazid and sensitive to rifampicin). The prevalence of isoniazid resistance and isoniazid mono-resistance was low. Only children < 15 years had higher prevalence of isoniazid resistance among those with rifampicin sensitive TB. Lastly, among patients with rifampicin sensitive TB, TB treatment outcomes were poorer among those with isoniazid resistant TB compared to those with isoniazid sensitive TB.

Our study had a number of strengths which included use of routine programmatic data, hence reflecting the ground realities of the national TB programme. Second, about three-quarters of the bacteriologically-confirmed TB patients underwent CDST, regardless of rifampicin resistance pattern and hence enabling estimation of INH prevalence particularly among those with rifampicin sensitive TB patients. Third, we adhered to the Strengthening the Reporting of Observational Studies in Epidemiology (STROBE) guidelines for reporting observational studies [16]. Our study was not without limitations. First, half of the bacteriologically confirmed cases with sputum samples received at the NTBRL had no culture growth and there were also differences in the distribution of patient characteristics between specimens with- and without- culture growth hence limiting inference of study findings. Second, there were also missing data for key study variables such as type of patient, site of TB and treatment outcomes of TB.

There are a number of reasons for gaps in receipt of sputum samples for CDST. Whilst it is recommended to collect two spot sputum samples, two hours apart for all presumptive TB patients, often only one sample is collected. The second sample is collected only among those detected with MTB upon following them up for treatment initiation in the field. Thus, the field workers fail to collect the samples among the patients lost-to follow-up prior to treatment. Also, frequent changes in guidelines on CDST and lack of communication to Gene Xpert sites could be other potential reasons for low rate of receipt of samples at the NTBRL.

The observed poor culture growth may be due to the decontamination process prior culture which might kill viable bacilli. Furthermore, there may have been a high proportion of salivary specimens which have been shown to yield culture negative results despite MTB being detected on Xpert assay [17]. The Xpert assay may also detect DNA from dead bacilli which will not show growth on culture media [18]. The volume of sputum submitted may have also been lower than the recommended 3 to 5 mL and hence affecting sputum quality. It is also plausible that the follow-up sputum that was submitted to the NTBRL among those with no culture growth were collected after the patients had been initiated on TB treatment. The mycobacterium growth rate has been shown to decline over time after commencing first-line anti-TB treatment when compared with Xpert MTB/RIF assay results [19,20].

The prevalence of isoniazid resistance observed in our study was lower compared to the 7.3% reported in other settings with high HIV prevalence and also significantly lower than the global prevalence of 13.9% and 44.9% reported in Eastern Europe [8]. Other studies have reported increases in isoniazid resistance in high HIV burden countries potentially due to scale-up of isoniazid preventive therapy (IPT) among PLHIV [9]. Zimbabwe has shown low rates despite high IPT coverage rates among PLHIV since the inception of IPT in ART care settings from 2011 onwards [21]. Although, IPT was scaled up there might be a lag time delay for developing the isoniazid resistance and circulation of strains with resistance among TB patients. Thus, there is need for monitoring the trends in proportion with isoniazid resistance over the coming years. This is by either implementing routine screening of INH resistance in the programme using routinely collected data for all bacteriologically confirmed TB patients or through conducting drug resistant surveys frequently.

Of note was that isoniazid resistance was predominantly higher among children < 15 years old and those who presented with new TB. The previous studies have not reported such high rates among children [22]. The reasons for the high rate seen among children in the current study are not known. However, it is cause for concern as children are most likely to have primary infection from adult contacts indicating the active circulation of isoniazid resistant TB strains. Thus, the future studies need to re-assess the association and if such association exists, the studies have to explore the potential reasons for such association.

Similar to evidence from previous studies, the current study also showed poor treatment outcomes among rifampicin sensitive TB patients with isoniazid resistance compared to those without resistance [9,22] though there was no statistically significant difference between the two groups in terms of death rates. This might be due to lower sample size for comparing the treatment outcomes.

The study has a few implications and recommendations. With poor treatment outcomes among isoniazid resistant TB patients, there is need for identifying such patients for provision of individualized regimens to improve treatment outcomes. The existing gaps and deficiencies in CDST for assessing isoniazid resistance has to be fixed. The recommended two spot sputum samples have to be collected on presentation of presumptive TB patients. The register with details on dispatch of the second sample for CDST and results of CDST has to be maintained for all the patients with MTB detected on Xpert assay. This will enable the programme managers to monitor the activity. There is need for monitoring of sputum quantity and quality among sputum samples submitted to the NTBRL for CDST in order to improve the proportion of samples with successful culture growth [23].

Alternatively, the point of care genotypic test like Abbott RealTime MTB RIF/INH Resistance (Abbott-RIF/INH) or whole genome sequencing (WGS) can be adopted for detection of INH resistance. The point-of-care tests will provide the results on isoniazid results instantly during diagnosis of TB and can aid in selecting appropriate treatment regimens. The genotypic tests are better options in high HIV burden settings with paucibacillary TB as large numbers of samples subjected to CDST fail to show culture growth and eventually go unassessed for isoniazid resistance.

With poor treatment outcomes among isoniazid TB patients, there is need for adopting a six month regimen course composed of rifampicin (R), ethambutol (E), pyrazinamide (Z) and levofloxacin (Lfx) (accronymed Hr-TB) as per WHO guidelines [7]. However, this will require allocation of additional resources and training of existing staff on the new regimen.

## Conclusion

The prevalence of isoniazid resistant TB was low compared to neighbouring countries with high burden of TB-HIV. However, Zimbabwe should consider reviewing treatment guidelines for isoniazid mono-resistant TB due to the observed poorer outcomes. Further investigations should be done to establish the reason why the half of the bacteriologically confirmed samples failed to grow on culture media. There is also need to identify reasons why a quarter of samples were not received at the culture laboratory since it might be affecting even the rifampicin resistant cases a well. Lastly the overall adverse treatment outcomes among those with isoniazid resistant TB add to the body of literature which advocate for a change in the standard approach of treating isoniazid mono-resistant TB cases.

## Supplementary Material

1

## Figures and Tables

**Table 1.** Socio-demographic and clinical characteristics of bacteriologically-confirmed TB patients in Bulawayo City, Zimbabwe (March 2017-December 2018).

Variable	n (%)
**Gender**	
Male	1,314 (60.8)
Female	846 (39.2)
**Age (in years)**	
< 15	45 (2.1)
15–24	267 (12.4)
25–34	609 (28.2)
35–44	603 (27.9)
45–54	304 (14.1)
55+	228 (10.6)
Not recorded	104 (4.8)
**Type of TB patient**	
New	2,031 (94.0)
Retreatment	67 (3.1)
Not recorded	62 (2.9)
**Site of TB**	
Pulmonary TB	2,017 (93.4)
Extra-pulmonary TB	9 (< 1)
Not recorded	134 (6.2)
**Genotypic rifampicin resistance pattern**	
Rif −ve	1,998 (92.5)
Rif +ve	153 (7.1)
Rif Indeterminate	9 (< 1)
**HIV status:**	
Negative	370 (17.1)
Positive	976 (45.2)
Not recorded	814 (37.7)
**On ART**	
Yes	741 (75.9)
Not recorded	235 (24.1)
**Total**	**2,160 (100)**

Rif +ve = Rifampicin resistance detected, Rif −ve = Rifampicin sensitive; HIV = Human immunodeficiency virus; ART = Antiretroviral therapy.

**Table 2.** Characteristics associated with isoniazid mono-resistance among bacteriologically-confirmed TB patients with culture growth among specimens received at the NTBRL from Bulawayo City, Zimbabwe (March 2017-December 2018).

Variable	N	INH mono-resistance^ǂ^, n (%)	PR (95% CI)	aPR (95% CI)^₸^
**Total**	**610**	**34 (5.6)**	**-**	**-**
**Gender**				
Male	405	23 (5.7)	Reference	Reference
Female	205	11 (5.4)	0.94 (0.47–1.90)	1.05 (0.52–2.12)
**Age category (in years):**				
< 15	12	3 (25.0)	**4.07 (1.29–12.86)**	**3.93 (1.24–12.45)**
15–24	93	0 (0)	-	
25–34	163	10 (6.1)	Reference	Reference
35–44	176	15 (8.5)	1.39 (0.64–3.00)	1.41 (0.65–3.06)
45–54	85	3 (3.5)	0.58 (0.16–2.03)	0.58 (0.17–2.06)
55+	57	2 (3.5)	0.57 (0.13–2.53)	0.54 (0.12–2.44)
Not recorded	24	1 (4.2)	0.68 (0.09–5.07)	0.64 (0.09–4.80)
**Type of TB patient:**				
New	575	34 (5.9)	Reference	Reference
Retreatment	32	0 (0)	-	-
Not recorded	3	0 (0)	-	-
**Site of TB**				
Pulmonary TB	594	34 (5.7)	Reference	Reference
Extra-pulmonary TB	2	0 (0)	-	-
Not recorded	14	0 (0)	-	-
**HIV & ART status**				
HIV −ve	149	8 (5.4)	Reference	Reference
HIV +ve, on ART	260	13 (5.0)	0.93 (0.40–2.19)	0.74 (0.31–1.76)
HIV +ve, ART status unknown	60	2 (3.3)	0.62 (0.14–2.84)	0.48 (0.11–2.21)
HIV status unknown	141	11 (7.8)	1.45 (0.60–3.51)	1.20 (0.51–2.84)

NTBRL = National TB Reference Laboratory; INH = Isoniazid; PR = Prevalence Ratio; APR = multivariate-adjusted Prevalence Ratio; CI = Confidence Interval; TB = Tuberculosis; HIV = Human Immunodeficiency Virus; HIV +ve = HIV positive; HIV−ve = HIV negative; ART = antiretroviral therapy;

ǂ Isoniazid mono-resistance- Resistant to isoniazid but sensitive to rifampicin;

₸ Log binomial model with all the exposure variables included.

**Table 3.** TB treatment outcomes among bacteriologically-confirmed TB patients with rifampicin sensitive TB stratified by isoniazid susceptibility pattern from Bulawayo City, Zimbabwe, (March 2017 – December 2018).

TB treatment outcome	Total		INH resistance status				P-value ǂ
			Resistance		Sensitive		
	n	(%)	n	(%)	n	(%)	
***Total***	**604**	**(100)**	**34**	**(100)**	**570**	**(100)**	
*Treatment Success*	*517*	(85.6)	25	(73.5)	492	(86.3)	***0.039***
Cured	477	(80.0)	24	(70.6)	453	(79.5)	0.217
Treatment completed	40	(6.6)	1	(2.9)	39	(6.8)	0.718
*Adverse outcome*	*87*	(14.4)	9	(26.5)	78	(13.7)	***0.039***
Died	42	(7.0)	5	(14.7)	37	(6.5)	0.078
Failure	4	(< 1)	0	(0)	4	(< 1)	-
LTFU	23	(3.8)	1	(2.9)	22	(3.9)	0.999
Not evaluated	18	(3.0)	3	(8.8)	15	(2.6)	0.074

TB = Tuberculosis; INH = Isoniazid; LTFU = loss to follow-up;

ǂ All p-values were determined using the Fischer’s Exact test excluding the p-value for comparisons for the proportions with “cured TB” and those with “TB treatment success” or “adverse outcome” where the Chi Square test was used;

₸ 6 patients were excluded in this analysis since they were not notified on TB treatment in Bulawayo City despite being diagnosed with TB.
