# Supplementary material for: Prevalence, risk factors and treatment outcomes of isoniazid resistant TB in Bulawayo city, Zimbabwe: A cohort study
Source: J Infect Dev Ctries. Author manuscript; Available in PMC 2021 Dec 9. (PMC8655986; doi:10.3855/jidc.12319)
Supplement: 1 [file NIHMS1760335-supplement-1.pdf]

Annex – Supplementary Items

Supplementary Table 1. Comparison of demographic and clinical characteristics with and without receipt sample at the NTBRL for CDST among bacteriologically-confirmed TB patients in Bulawayo City, Zimbabwe (March 2017-December 2018).

Variable	Receipt of sample at NTBRL, n (%)		P-value
	No	Yes	
Total	548 (100)	1609 (100)	
**Gender**			
Male	332 (60.6)	982 (60.9)	0.89
Female	216 (39.4)	630 (39.1)	
**Age category (in years):**			
< 15	9 (1.6)	36 (2.2)	0.532
15-24	65 (11.9)	202 (12.5)	
25-34	172 (31.4)	437 (27.1)	
35-44	150 (27.4)	453 (28.1)	
45-54	75 (13.7)	229 (14.2)	
55+	50 (9.1)	178 (11.0)	
Not recorded	27 (4.9)	77 (4.8)	
**Type of TB patient:**			
New	526 (96.0)	1505 (93.4)	0.067
Retreatment	13 (2.4)	54 (3.4)	
Not recorded	9 (1.6)	53 (3.3)	
**Site of TB**			
Pulmonary TB	523 (95.4)	1494 (92.7)	**0.013**
Extra-pulmonary TB	4 (< 1)	5 (< 1)	
Not recorded	21 (3.8)	113 (7.0)	
**Genotypic rifampicin resistance pattern**			
Rif -ve	512 (93.4)	1,486 (92.2)	0.170
Rif +ve	32 (5.8)	121 (7.5)	
Rif Indeterminate	4 (< 1)	5 (< 1)	
**HIV and ART status**			
HIV-ve	108 (19.7)	262 (16.3)	**< 0.001**
HIV +ve, on ART	215 (39.2)	526 (32.6)	
HIV +ve, ART status unknown	57 (10.4)	178 (11.0)	
HIV status unknown	168 (30.7)	646 (40.1)	

TB = Tuberculosis; NTBRL = National Tuberculosis Reference Laboratory; CDST = Culture and Drug Susceptibility Test; Rif +ve = Rifampicin resistance detected, Rif -ve = Rifampicin sensitive; HIV = Human immunodeficiency virus; ART = Antiretroviral therapy.

Supplementary Table 2. Comparison of demographic and clinical characteristics with and without culture growth on CDST among bacteriologically-confirmed TB patients in Bulawayo City, Zimbabwe (March 2017-December 2018).

Variable	N	Culture growth, n (%)		P-value
		No	Yes	
Total	1,612	869 (100)	743 (100)	
**Gender**				
Male	982	493 (56.7)	489 (65.8)	**< 0.001**
Female	630	376 (43.3)	254 (34.2)	
**Age category (in years):**				
< 15	36	23 (2.7)	13 (1.75)	**0.005**
15-24	202	87 (10.0)	115 (15.5)	
25-34	437	231 (26.6)	206 (27.7)	
35-44	453	245 (28.2)	208 (28.0)	
45-54	229	125 (14.4)	104 (14.0)	
55+	178	107 (12.3)	71 (9.6)	
Not recorded	77	51 (5.9)	26 (3.5)	
**Type of TB patient:**				
New	1,505	805 (92.6)	700 (94.2)	**< 0.001**
Retreatment	54	16 (1.8)	38 (5.1)	
Not recorded	53	48 (5.5)	5 (< 1)	
**Site of TB**				
Pulmonary TB	1,494	769 (88.5)	725 (97.6)	**< 0.001**
Extra-pulmonary TB	5	3 (< 1)	2 (< 1)	
Not recorded	113	97 (11.2)	16 (2.2)	
**HIV and ART status**				
HIV-ve	262	84 (9.7)	178 (24.0)	**< 0.001**
HIV +ve, on ART	526	219 (25.2)	307 (41.3)	
HIV +ve, ART status unknown	178	92 (10.6)	86 (11.6)	
HIV status unknown	646	474 (54.6)	172 (23.2)	

TB = Tuberculosis; CDST = Culture and Drug Susceptibility Test; Rif +ve = Rifampicin resistance detected, Rif -ve = Rifampicin sensitive; HIV- Human immunodeficiency virus; ART- Antiretroviral therapy.
